# On the *Neoechinorhynchus agilis* (Acanthocephala: Neoechinorhynchidae) complex, with a description of *Neoechinorhynchus ponticus* n. sp. from *Chelon auratus* in the Black Sea

**DOI:** 10.1051/parasite/2020044

**Published:** 2020-07-23

**Authors:** Omar M. Amin, Meysam Sharifdini, Richard A. Heckmann, Nataliya Rubtsova, Halima Jmii Chine

**Affiliations:** 1 Institute of Parasitic Diseases 11445 E. Via Linda # 2-419 Scottsdale 85259 AZ USA; 2 Department of Medical Parasitology and Mycology, School of Medicine, Guilan University of Medical Sciences 3363 Rasht Iran; 3 Department of Biology, Brigham Young University 1114 MLBM Provo 84602 UT USA; 4 University of Tunis El Manar, Faculty of Sciences of Tunis, Laboratory of Diversity, Management and Conservation of Biological Systems LR18ES06 Tunis Tunisia

**Keywords:** Acanthocephala, *Neoechinorhynchus agilis*, *N. personatus*, *N. yamagutii*, *N. ponticus* n. sp., species complex, *Mugil cephalus*, *Chelon auratus*, Mediterranean, Black Sea

## Abstract

We recognize four species in the *Neoechinorhynchus agilis* complex. We studied specimens of *Neoechinorhynchus* (*Hebesoma*) *personatus* Tkach, Sarabeev & Shvetsova, 2014 from *Mugil cephalus* in the Mediterranean Sea off Tunisia and in the Black Sea, and also specimens of *Neoechinorhynchus ponticus* n. sp. from *Chelon auratus* Risso in the Black Sea. Specimens from *M. cephalus* at both locations were similar. All structures of *N. ponticus* n. sp. were considerably smaller than those of *N. personatus*. Two other species of the *N. agilis* complex are recognized: *Neoechinorhynchus agilis* (Rudolphi, 1819) *sensu stricto* from various hosts in the Atlantic and the Mediterranean, and *Neoechinorhynchus yamagutii* Tkach, Sarabeev & Shvetsova, 2014 from *M. cephalus* and *Planiliza haematocheila* in the Pacific, especially the Sea of Japan. *Neoechinorhynchus dimorphospinus* Amin & Sey, 1996 from marine fish in the Persian Gulf and the Pacific Ocean off Vietnam may be a candidate for membership in the *N. agilis* complex. X-ray scans of gallium cut and intact hooks of *N. personatus* and *N. ponticus* showed differences in the mineral content of hooks with higher sulfur levels in smaller hooks and in hooks from specimens in the Black Sea compared to specimens from the Mediterranean. The relatively high genetic differences between *N. ponticus* n. sp. and other species of *Neoechinorhynchus* using a partial 18S rDNA dataset support its independent status. *Neoechinorhynchus ponticus* n. sp. and *N. personatus* have a common ancestor with species of *Neoechinorhynchus* collected from saltwater fish.

## Abstract (fr)

Nous reconnaissons quatre espèces dans le complexe *Neoechinorhynchus agilis*. Nous avons étudié des spécimens de *Neoechinorhynchus (Hebesoma) personatus* Tkach, Sarabeev & Shvetsova, 2014 de *Mugil cephalus* en mer Méditerranée au large de la Tunisie et en Mer Noire et également des spécimens de *Neoechinorhynchus ponticus* n. sp. de *Chelon auratus* Risso en Mer Noire. Les spécimens chez *M. cephalus* dans les deux endroits étaient similaires. Toutes les structures de *N. ponticus* n. sp. étaient considérablement plus petites que celles de *N. personatus*. Deux autres espèces du complexe *N. agilis* sont reconnues: *Neoechinorhynchus agilis* (Rudolphi, 1819) *sensu stricto* provenant de divers hôtes de l’Atlantique, y compris la Méditerranée, et *Neoechinorhynchus yamagutii* Tkach, Sarabeev & Shvetsova, 2014 de *M. cephalus* et *Planiliza haematocheila* dans le Pacifique, en particulier la Mer du Japon. *Neoechinorhynchus dimorphospinus* Amin & Sey, 1996 provenant de poissons marins du golfe Persique et de l’océan Pacifique au large du Vietnam pourrait être candidat à l’appartenance au complexe *N. agilis*. Les radiographies des crochets coupés au gallium et des crochets intacts de *N. personatus* et *N. ponticus* ont montré des différences dans la teneur en minéraux des crochets avec des niveaux de soufre plus élevés dans les hameçons plus petits et dans les hameçons des spécimens de la Mer Noire par rapport aux spécimens de la Méditerranée. Les différences génétiques relativement élevées entre *N. ponticus* n. sp. et d’autres espèces de *Neoechinorhynchus* utilisant un ensemble de données d’ADNr 18S partiel soutiennent son statut indépendant. *Neoechinorhynchus ponticus* n. sp. et *N. personatus* ont un ancêtre commun avec des espèces de *Neoechinorhynchus* prélevées sur des poissons d’eau salée.

## Introduction

Rudolphi [30] originally described the external morphology and proboscis receptacle of *Neoechinorhynchus agilis* (Rudolphi, 1819) Van Cleave, 1916 from 9 individuals taken from *Mugil cephalus* in Spezia, Italy without providing any measurements. Hamann [13] described *N. agilis* in detail but thought it had 6 cement glands. Van Cleave [36] described a syncytium cement gland containing 8 giant nuclei. Van Cleave [36, 37] suspected that *N. agilis* was restricted to the Mediterranean, but this is now known not to be. For example, species presumed to be *N. agilis* before the establishment of the species complex concept studies by Tkach et al. [35], Sarabeev et al. [31] and ours (this paper) were reported from *M. cephalus* in Taiwan [32], in the Indian Ocean near Tamil Nadu [17], and in Guyana [26], as well as in Japan, Scotland, and North America. Other observers including Linton [19–21], Van Cleave [36, 37], Meyer [23], Yamaguti [40, 41], Petrochenko [24], Gaevskaya et al. [10], and Tepe and Oguz [34] reported *N. agilis* from at least 10 species of fish in the Black Sea and the Mediterranean, among other locations off the coasts of Scotland, North America, and Japan. This species complex appears to be widely distributed in mullets from the Atlantic and Pacific oceans ([31, 35], among others). Altogether, descriptions by the above cited authors gave an extreme range of variation in taxonomically important traits. Van Cleave [37] provided a detailed description of the variability in “*N. agilis*” based on the examination of Rudolphi’s specimens and other materials from the Berlin Museum (Table IV of Van Cleave [37]).

None of the above authors reported on the relationship between the observed extreme variability and host species. Van Cleave [37] was unable to determine whether the “conflicting data represented individual variability within the species or resulted from inaccurate observations and erroneous identifications.” Tkach et al. [35] split *N. agilis* into three species that showed some overlap in measurements and that were collected from *M. cephalus* and other mugilids from various locations from the Mediterranean to Japan. These are *Neoechinorhynchus* (*Hebesoma*) *personatus* Tkach, Sarabeev & Shvetsova, 2014, *Neoechinorhynchus agilis sensu stricto*, and *Neoechinorhynchus yamagutii* Tkach, Sarabeev & Shvetsova, 2014. Gargouri et al. [12] and Tepe and Oguz [34] reported “*N. agilis*” from *C. auratus* in the Mediterranean off the Tunisian coast at the Bizerte Lagoon and off the Black Sea coast of Turkey, respectively. Observations were not available to correlate their morphometric data with host species.

We also provide chemical and molecular data to explain and clarify our findings. With the use of dual beam scanning electron microscopy, it was feasible to run energy dispersive X-ray analysis (EDXA) on both intact and gallium (LMIS) cut hooks. This is usually a qualitative analysis, not quantitative, for the attachment structures [5, 14, 15]. This technique was applied to the study of the *Neoechinorhynchus* complex. This study provides molecular profiles of *N. ponticus* n. sp*.* and *N. personatus* based on 18S rDNA gene. Furthermore, its phylogenetic relationships with other members of the genus *Neoechinorhynchus* are analyzed and discussed.

## Materials and methods

### Collections

Five hundred and twenty two individuals of 5 mugilid species of fish in the Ichkeul Lagoon off Northern Tunisia (37°10′00″ N, 9°40′00″ E) were sampled between March 2014 and September 2017 by gill net fishing. Fish were examined for helminths under a stereomicroscope shortly after capture. The specimens of acanthocephalans collected were first placed in distilled water at 4 °C until the proboscis was everted. Tunisian specimens of *N. personatus*, among other acanthocephalan species, from *M. cephalus* from this collection were deposited in the Harold W. Manter Laboratory (HWML) parasitology collection no. 102004. In the Black Sea at Zalizny Port (46°7′ N, 32°17′ E), individuals of *M. cephalus* and of *Chelon auratus* were examined in June, 2015. Specimens of *N. personatus* from *M. cephalus* were deposited in the HWML collections no. 102006 and those of *N. ponticus* from *C. auratus* in the HWML collection no. 102005.

### Methods for microscopical studies

Worms were punctured with a fine needle and subsequently stained in Mayer’s acid carmine, destained in 4% hydrochloric acid in 70% ethanol, dehydrated in ascending concentrations of ethanol (24 h each), and cleared in 100% xylene then in 50% Canada balsam and 50% xylene (24 h each). Whole worms were then mounted in Canada balsam. Measurements are in micrometers, unless otherwise noted; the range is followed by the mean values between parentheses. Width measurements represent maximum width. Trunk length does not include proboscis, neck, or bursa.

Microscope images were created using 10X and 40X objective lenses of a BH2 light Olympus microscope (Olympus Optical Co., Osachi-shibamiya, Okaya, Nagano, Japan) attached to an AmScope 1000 video camera (United Scope LLC, dba AmScope, Irvine, CA, USA), linked to an ASUS labtop equipped with an HDMI high definition multimedia interface system (Taiwan–USA, Fremont, CA, USA). Images from the microscope were transferred from the labtop to a USB and stored for subsequent processing on a computer.

### Scanning electron microscopy (SEM)

Samples of parasites that had been fixed and stored in 70% ethanol were processed following standard methods [18] which included critical point drying (CPD) in sample baskets and mounted on SEM sample mounts (stubs) using conductive double-sided carbon tape. Samples were coated with gold and palladium for 3 min using a Polaron #3500 sputter coater (Quorum (Q150 TES) www.quorumtech.com) establishing an approximate thickness of 20 nm. Samples were placed and observed in an FEI Helios Dual Beam Nanolab 600 (FEI, Hillsboro, OR, USA) Scanning Electron Microscope with digital images obtained in the Nanolab software system (FEI) and then transferred to a USB for future reference. Images were taken at various magnifications. Samples were received under low vacuum conditions using a 10 kV, spot size 2 0.7 Torr using a GSE detector.

#### X-ray microanalysis, energy dispersive X-ray analysis

Standard methods were used for preparation, similar to the SEM procedure. Specimens were examined and positioned with the above SEM instrument which was equipped with a Phoenix energy-dispersive X-ray analyzer (FEI). X-ray spot analysis and live scan analysis were performed at 16 kV with a spot size of 5 and results were recorded on charts and stored with digital imaging software attached to a computer. The TEAM* (Texture and Elemental Analytical Microscopy) software system (FEI) was used. The data included weight percent and atom percent of the detected elements following correction factors.

### Ion sectioning of hooks

A dual-beam SEM with a gallium (Ga) ion source (GIS) was used for the liquid ion metal source (LIMS) part of the process. The hooks of the acanthocephalans were sectioned using a probe current between 0.2 nA and 2.1 nA, according to the rate at which the area is cut. The time of cutting was based on the nature and sensitivity of the tissue. Following the initial cut, the sample also goes through a milling process to obtain a smooth surface. The cut was then analyzed for chemical ions with an electron beam (Tungsten) to obtain an X-ray spectrum. The intensity of the GIS was variable due to the nature of the material being cut.

### DNA extraction and PCR amplification

Total genomic DNA was extracted from 3 adult worms of *N. ponticus* n. sp. and 4 of *N. personatus* using a Qiagen DNeasy blood and tissue kit (Qiagen Inc., Valencia, CA, USA), according to the manufacturer’s instructions. Partial nuclear small subunit ribosomal DNA (18S rDNA) was amplified by PCR reactions in 30 μL volumes containing 2× red PCR premix (Ampliqon, Odense, Denmark), 20 pmol of each primer, and 3 μL of isolated DNA. Forward primer (5′ – AGATTAAGCCATGCATGCGTAAG – 3′) and reverse primer (5′ – ACCCACCGAATCAAGAAAGAG – 3′) were used for the amplification of the partial fragment of the nuclear 18S rDNA gene [7]. PCR conditions were: 95 °C for 5 min followed by 35 cycles of 95 °C for 30 s, 61 °C for 30 s, 72 °C for 60 s, and 72 °C for 7 min as a final extension. Finally, the PCR products were electrophoresed on a 1.5% agarose gel and visualized with a UV transilluminator (Vilber Lourmat, Collégien, France).

### Sequencing and phylogenetic analysis

All PCR products were sequenced on an ABI 3730 automatic sequencer (Applied Biosystems, Foster City, CA, USA) in both directions using the same PCR primers. The obtained sequences were edited and analyzed using Chromas v.2.01 (Technelysium Pty Ltd., Brisbane, Queensland, Australia) and BioEdit software v.7.0.9. The sequences were compared with available data in GenBank using the BLAST algorithm (http://www.ncbi.nlm.nih.gov/). The nucleotide sequences of the 18S rDNA gene from this study were submitted to the GenBank database (Accession Numbers: MT020789 – MT020791 for *N. ponticus* n. sp. isolates and MT020792 – MT020795 for *N. personatus* isolates).

A phylogenetic tree was constructed using the maximum-likelihood algorithm and Tamura-3-parameter model in MEGA7 software (http://www.megasoftware.net/). A bootstrap value with 1000 replications was also implemented to evaluate the reliability of the tree topologies. Furthermore, genetic distances were calculated with the maximum composite likelihood model using MEGA7 software. The sequences used for the phylogenetic analysis are listed in Table 1.

**Table 1:** Acanthocephalan species represented in the phylogenetic analysis with their host species, GenBank accession numbers, and locations.

Species	Host	GenBank acc. no. 18S rDNA	Location
*Neoechinorhynchus personatus* Tkach, Sarabeev & Shvetsova, 2014	*Mugil cephalus*	MT020792–MT020795	Tunisia
*Neoechinorhynchus ponticus* n. sp.	*Liza aurata*	MT020789–MT020791	Ukraine
*Neoechinorhynchus personatus* Tkach, Sarabeev & Shvetsova, 2014	*Mugil cephalus*	MN149068, MN149069 and MN149071	Ukraine
*Neoechinorhynchus agilis* (Rudolphi, 1819)	*Chelon labrosus*	MN148893 and MN148895	Spain
*Neoechinorhynchus* sp*.*	*Siganus fuscescens*	HM545898	China
*Neoechinorhynchus dimorphospinus* (Amin & Sey, 1996)	*Liza subviridis*	MK510080	Thailand
*Neoechinorhynchus yamagutii* Tkach, Sarabeev and Shvetsova, 2014	*Mugil cephalus*	MN149220	Russia
*Neoechinorhynchus buttnerae* (Golvan, 1956)	Not available	MK249749	Brazil
*Neoechinorhynchus crassus* (Van Cleave, 1919)	*Capoeta aculeata*	KU363971	Iran
*Neoechinorhynchus pseudemydis* (Cable & Hopp, 1954)	*Capoeta aculeata*	KU363973	Iran
*Neoechinorhynchus cylindratus* (Van Cleave, 1913)	*Micropterus salmoides*	MF974925	USA
*Neoechinorhynchus beringianus* (Mikhailova & Atrashkevich, 2008)	*Pungitius pungitius*	KF156875	Russia
*Neoechinorhynchus saginata* (Van Cleave & Bangham, 1949)	Not available	AY830150	Not available
*Neoechinorhynchus simansularis* (Roytman, 1961)	*Salvelinus alpinus*	KF156877	Russia
Outgroup			
*Echinorhynchus gadi*	*Salvelinus malma*	KF156880	Russia
*Corynosoma enhydri*	*Enhydra lutris*	AF001837	Na
*Bolbosoma caenoforme*	*Salvelinus malma*	KF156879	Russia

## Results

Of 522 individuals of 5 mugilid species of fish examined in the Ichkeul Lagoon off the Mediterranean in Northern Tunisia between March, 2014 and September, 2017, 66 of 117 individuals of *M. cephalus* were found to be infected with 564 acanthocephalans among them (about 200) specimens of *N. personatus* in June, 2014. Other mugilid species examined included *Chelon auratus*, *Chelon ramada* Risso, *Chelon saliens* Risso, and *Chelon labrosus* Risso. Acanthocephalans were mostly found in the anterior intestine except in one case where 49 fully mature normal adult acanthocephalans were found in the body cavity of one individual *M. cephalus* in June, 2014. In the Black Sea at Zalizny Port, all 7 examined individuals of *M. cephalus* were infected with 82 specimens of *N. personatus* (range 1–27; mean 11.7) and all 4 individuals of *C. auratus* were infected with 36 specimens of *N. ponticus* n. sp. (range 3–18; mean 9.0).

### Neoechinorhynchus (Hebesoma) personatus Tkach, Sarabeev, Shvetsova, 2014 (Figs. 1, 3, 5–7, 9, 11–30)

Type and Tunisian host: *Mugil cephalus* Linnaeus (Mugilidae).

**Figures 1–10:**
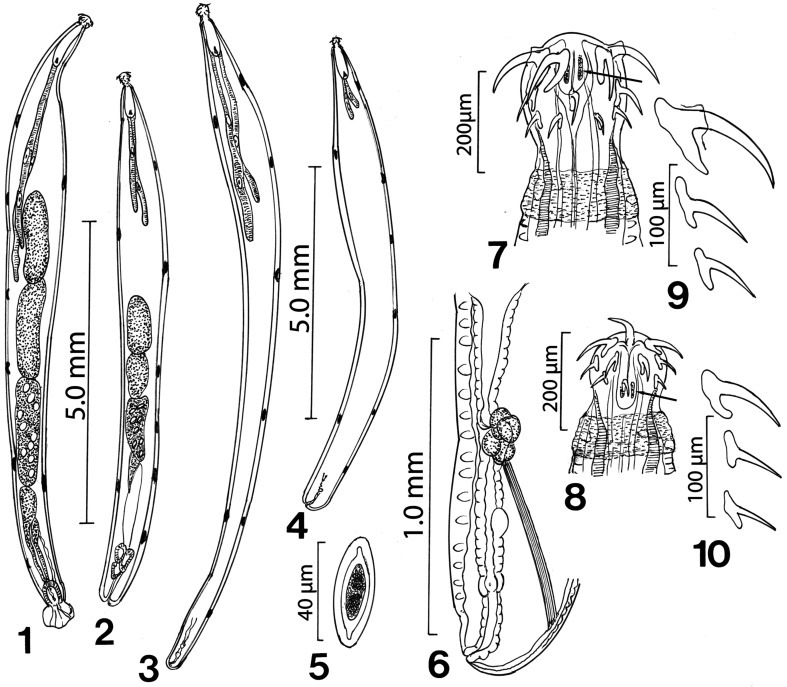
A comparison between specimens of *Neoechinorhynchus personatus* from *Mugil cephalus* in the Mediterranean Sea off Tunisia and from the Black Sea (Figs. 1, 3, 5–7, 9) and specimens of *Neoechinorhynchus ponticus* n. sp. from *Chelon auratus* from the Black Sea (Figs. 2, 4, 8, 10). **1.** Adult male specimen of *N. personatus* from *M. cephalus* in Tunisia*.* Note the extension of the unequal lemnisci to the anterior testis and the number of the giant nuclei in the cement gland. **2.** A smaller male specimens of *N. ponticus* from *C. auratus*, in the Black Sea, drawn to same scale. Note the shorter lemnisci and smaller reproductive system. **3.** A female specimen of *N. personatus* with long unequal lemnisci, near terminal gonopore, and 6 dorsal and 2 ventral giant subcutaneous nuclei. **4.** A smaller adult female specimen of *N. ponticus* n. sp. from *C. auratus* in the Black Sea, drawn to same scale as Fig. 3. Note small lemnisci. Eggs and ovarian balls not shown. **5.** A ripe egg from a gravid female specimen of *N. personatus* from *M. cephalus*. **6.** A female reproductive system of a specimen of *N. personatus* from *M. cephalus* which is comparable to that of *N. ponticus* from *C. auratus*. Note the prominent ligament strand connecting anterior end of uterus at uterine glands with posterior body wall near the vagina dorsally. **7.** Proboscis of a male specimen of *N. personatus* from *M. cephalus* in Tunisia. Note the 2 giant nuclei in the prominent apical organ (arrow), and the anterior trunk ring. **8.** The smaller proboscis of a male specimen of *N. ponticus* n. sp. from *C. auratus,* in the Black Sea drawn to same scale as Figure 7. An arrow points to the giant nuclei of the apical organ. **9**. A high magnification of one row of hooks from the proboscis of a male specimen of *N. personatus* from *M. cephalus.***10.** A high magnification of one row of the smaller hooks from the proboscis of a male specimen of *N. ponticus* from *C. auratus*. drawn to same scale as the hooks in Fig. 9.

**Figures 11–16:**
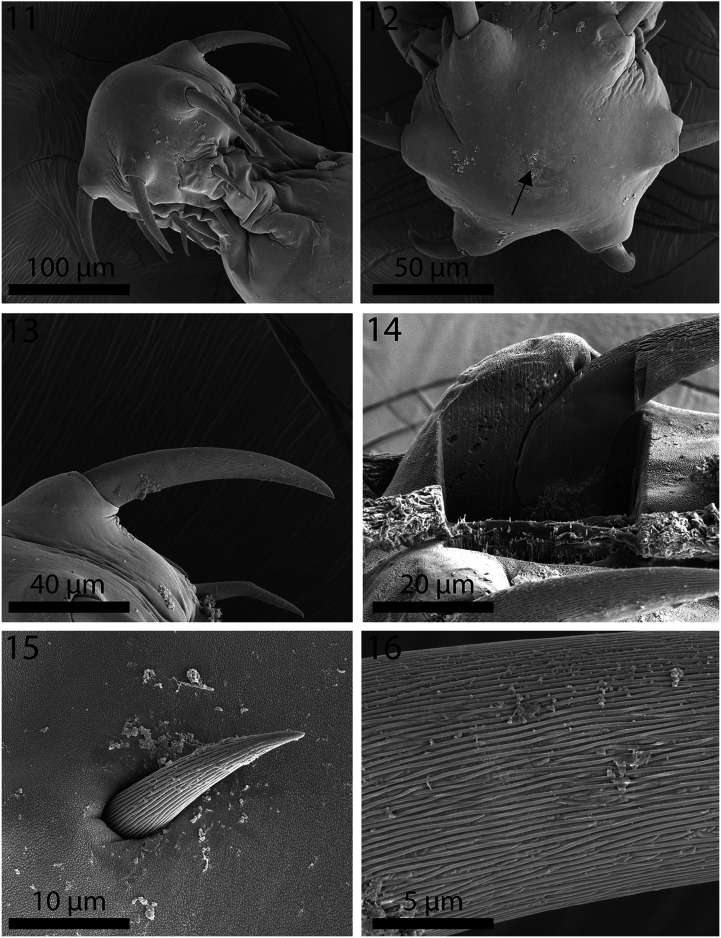
SEM of specimens of *Neoechinorhynchus personatus* from *Mugil cephalus* in the Mediterranean Sea off Tunisia. **11.** Proboscis of a male specimen showing the large anterior hooks and the delineation of the neck. **12.** An apical view of a proboscis showing the pore of the apical organ (arrow). **13.** The magnified anterior and middle hooks of the proboscis shown in Fig. 11. **14.** A partially Gallium cut anterior hook showing its thin cortical layer and dense core, and its articulation vs. the root of the same core density. **15.** The longitudinal serrations on the posterior hook are evident. **16.** A magnified view of an anterior hook depicting the pattern of the serrations.

Type-locality: Azov-Black Sea region (Tkach, Sarabeev & Shvetsova, 2014).

Other localities: Mediterranean Sea (Tkach, Sarabeev & Shvetsova, 2014).

Present localities: Ichkeul Lagoon off the Mediterranean in Northern Tunisia **(**37°10′ N, 9°40′ E) and the Black Sea at Zalizny Port, Ukraine (46º7′ N, 32°17′ E).

Site of infection: Intestine and peritoneal cavity.

Voucher specimens: Harold W. Manter Lab collection nos. HWML 102004 (from *M. cephalus* off Tunisia), and HWML 102006 (from *M. cephalus* from the Black Sea).

The following redescription is based on the microscopic examination of 26 specimens (9 males, 17 females) from *M. cephalus* in the Black Sea and 16 specimens (8 males, 8 females) off the Tunisian coast, and on 6 more specimens of each collection examined by SEM. Additional specimens were used for molecular analysis. The redescription addresses mostly qualitative features that apply to the two comparable populations of *N. personatus* studied in these two geographical locations, presenting a number of features not noted in the original description. The specimens from the Black Sea and the Mediterranean were collected in the same month, June, 2014 and 2015 (in the Black Sea). Morphological and morphometric data are presented in Table 2. Biochemical, EDXA and molecular analysis are addressed separately, below.

**Table 2:** Morphometric comparisons among species of *Neoechinorhynchus* from *Mugil cephalus* and *Chelon auratus* in the Mediterranean and the Black Sea in our collections.

*Neoechinorhynchus* sp.	*N. personatus*	*N. personatus*	*N. ponticus* n. sp.
Host	*Mugil cephalus*	*Mugil cephalus*	*Chelon auratus*
Locality	Tunis, Mediterranean	Black Sea	Black Sea
*Males*	*n* = 8	*n* = 9	*n* = 9
Trunk (mm)	9.55–10.07 (9.80) × 0.87–1.10 (1.0)*	7.25–10.25 (9.00) × 0.53–0.92 (0.70)	7.87–8.75 (8.44) × 0.67–1.00 (0.85)
Proboscis	182–205 (190) × 205–225 (213)	162–218 (200) × 200–242 (224)	100–145 (127) × 135–175 (151)
Hook length			
Ant.	107–147 (123)	112–130 (123)	77–87 (82)
Mid.	70–85 (75)	65–85 (78)	40–57 (45)
Post.	53–71 (60)	57–72 (66)	30–40 (34)
Receptacle	728–1,019 (882) × 218–281 (253)	780–936 (852) × 187–291 (221)	603–728 (653) × 135–187 (157)
Lemnisci (mm)			
Short	3.12–4.25 (3.70) × 0.13–0.19 (0.15)	2.86–3.26 (3.06) × 0.10–1.3 (1.2)	1.73–2.75 (2.14) × 0.10–0.15 (0.14)
Long	3.12–4.67 (3.86) × 0.16–0.19 (0.17)	3.57–4.05 (3.81) × 0.11–0.19 (0.15)	2.03–2.81 (2.41) × 0.12–0.19 (0.14)
Anterior testis (mm)	1.25–1.70 (1.44) × 0.35–0.50 (0.45)	0.70–1.50 (1.11) × 0.32–0.45 (0.38)	0.25–1.00 (0.70) × 0.35–0.50 (0.44)
Posterior testis (mm)	1.25–1.75 (1.49) × 0.25–0.50 (0.43)	1.00–1.45 (1.19) × 0.30–0.45 (0.35)	0.50–0.87 (0.63) × 0.40–0.50 (0.44)
Cement gland (mm)	1.67–2.67 (2.09) × 0.37–0.50 (0.45)	1.00–1.77 (1.29) × 0.22–0.40 (0.31)	0.75–1.50 (0.96) × 0.45–0.52 (0.49)
Cement reservoir	450–625 (560) × 225 – 350 (300)	375–520 (465) × 250–312 (270)	375–500 (431) × 253–375 (310)
Saefftigen’s pouch (mm)	Obscured	Obscured	0.77–1.25 (1.08) × 0.12–0.30 (0.21)
Bursa (mm)	0.42–1.20 (0.72) × 0.40–0.52 (0.47)	0.50–1.25 (0.94) × 0.20–0.42 (0.30)	–
*Females*	n=8	n=17	n=10
Trunk (mm)	11.00–14.75 (13.65) × 0.77–1.27 (1.05)	8.00–21.25 (14.48) × 0.50–1.22 (0.91)	8.37–10.75 (9.73) × 0.57–0.95 (0.82)
Proboscis	162–200 (184) × 205–230 (217)	187–239 (215) × 208–250 (231)	112–142 (129) × 132–162 (152)
Hook length			
Ant.	110–137 (124)	105–142 (123)	75–132 (90)
Mid.	62–80 (71)	75–90 (81)	40–77 (50)
Post.	60–67 (64)	62–77 (68)	32–67 (40)
Receptacle	832–1,040 (910) × 218–333 (260)	707–988 (856) × 198–364 (273)	634–780 (718) × 156–208 (179)
Lemnisci (mm)			
Short	3.70 × 0.15	4.80 × 0.17	2.00–2.75 (2.46) × 0.10–0.15 (0.13)
Long	3.90 × 0.17	4.86 × 0.17	2.50–3.75 (3.33) × 0.12–0.17 (0.14)
Eggs	45–50 (48) × 10–12 (10)	35–47 (40) × 10–15 (12)	–
Reproductive syst. (mm)	1.25–1.56 (1.44)	0.96–1.67 (1.29)	0.73–1.25 (1.09)

Range (mean) in μm unless otherwise stated.

#### Redescription

**Figures 17–22:**
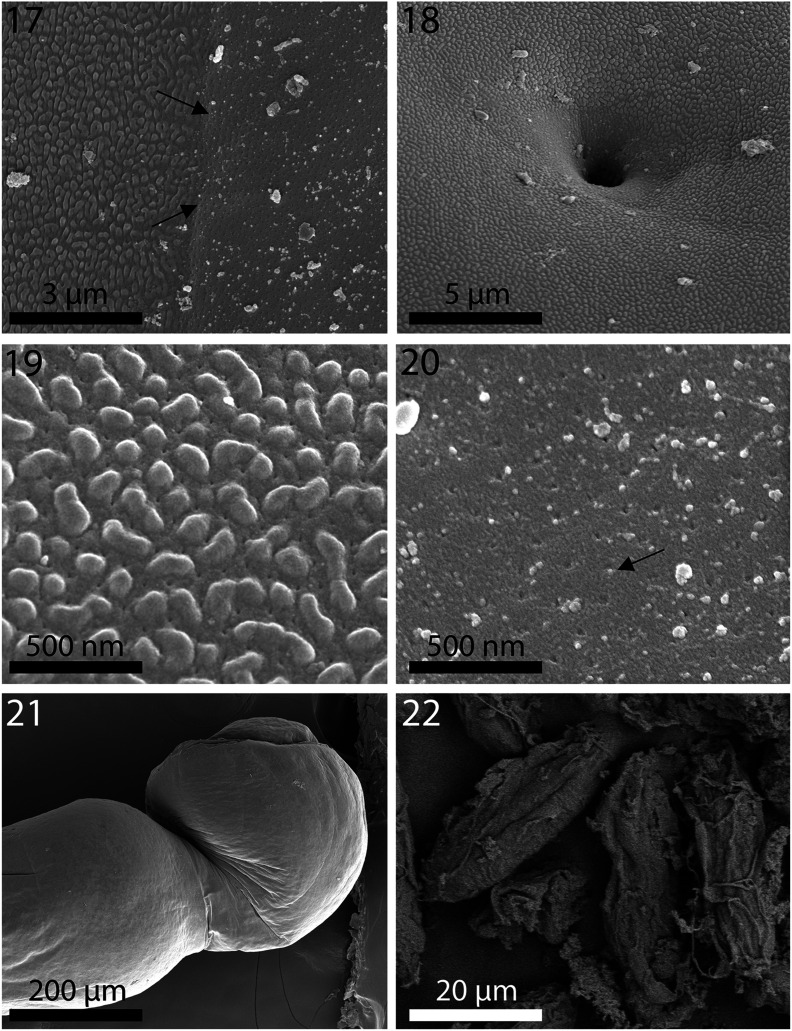
SEM of specimens of *Neoechinorhynchus personatus* from *Mugil cephalus* in the Mediterranean Sea off Tunisia. **17.** The separation line (arrows) between the pebbled neck surface (left) and the anterior trunk with micropores of a specimen. **18.** A sensory pore in the neck of a specimen showing the pebbled surface of the neck and the non-pebbled depression around the pore. **19.** A magnified view of the neck surface depressions. **20.** Micropores (arrow) in the anterior trunk of a specimen. **21.** The bursa of a male specimen showing no visible ornamentation or pores. **22.** Eggs.

**Figures 23–30:**
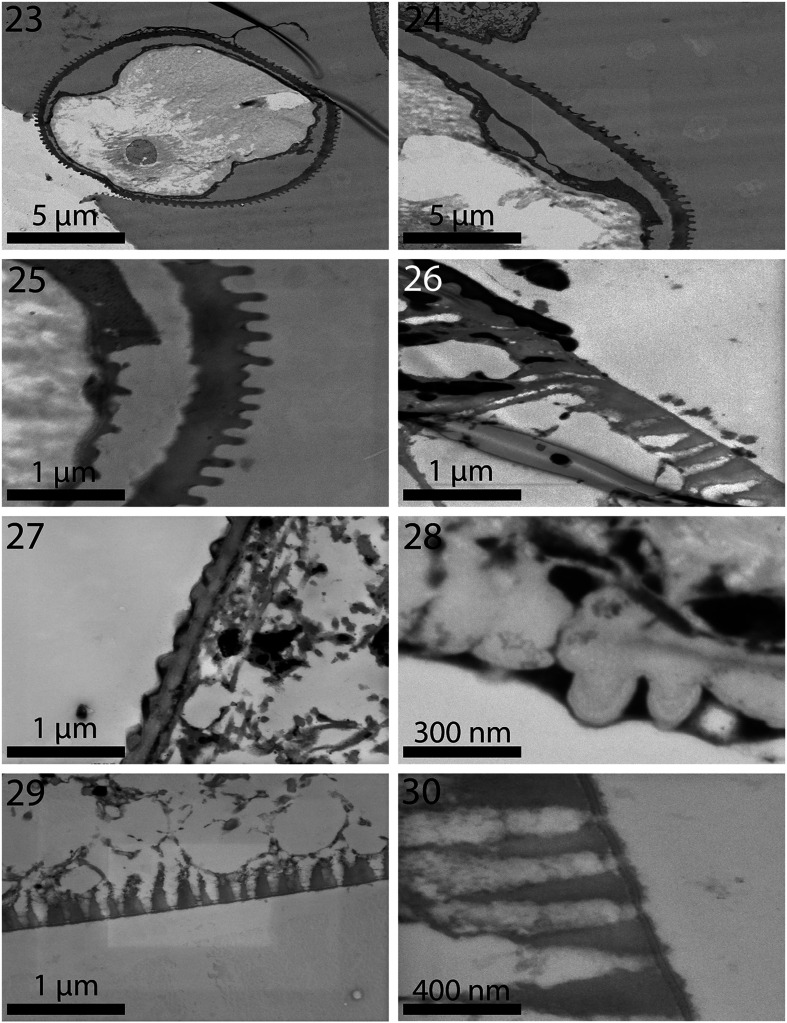
TEM sections of specimens of *Neoechinorhynchus personatus* from *Mugil cephalus* in the Mediterranean Sea off Tunisia. **23.** Cross section of a hook. Note the serrated (corrugated) outer layer and the solid inner core with an internal tube. **24.** Higher magnification of pert of the hook in Fig. 23 showing the outer serrated layer. **25.** Outermost layer of the hook showing detail toothed serrations. **26.** Transition between the neck (top) and trunk (bottom with micropore tubules) of a worm. **27.** Outer tegument layer of the neck with a knobby (pebbled) surface. No visible micropore channels here. **28.** A high magnification of the neck tegument in Fig. 27. Note distinct outer layer and myofibrils beneath the tegument. **29.** Part of the trunk tegument of a worm showing micropore channels. **30.** A higher magnification of a section of the tegument in Fig. 29 showing detail of the micropore channels.

*General*: With characters of the genus *Neoechinorhynchus* and the subgenus *Hebesoma* as designated by Amin [1] (Neoechinorhynchidae). Medium-sized worms. Shared structures larger in females than in males. Trunk cylindrical, somewhat enlarged anteriorly, gradually tapering at both ends (Figs. 1, 3). Body wall with electron dense micropores ducting into subcutaneous layer with multiple branching (Figs. 20, 26, 29, 30). Neck prominent with paired sensory pores and elevated pebble-like protrusions and paired sensory pores (Figs. 17–19, 26–28). Trunk with prominent reticular lacunar system usually manifesting as regular alternating bands, and with 6–7 (usually 6) dorsal and 1–2 (usually 2) ventral hypodermal giant nuclei. Muscular ring at anterior trunk variably distinguished but usually prominent (Fig. 7). Proboscis cylindrical, truncated, widest anteriorly, relatively but consistently wider than long, with prominent apical organ having 2 conspicuous nuclei at its distal end (Figs. 7, 11, 12). All hooks serrated longitudinally (Figs. 13–16, 23–25), rooted, most robust with longest roots anteriorly (Fig. 14), smallest posteriorly, equal in length in each circle. Hook roots prominent but markedly shorter than blades with anteriorly directed manubria. Manubria least prominent in largest anterior hooks (Fig. 9). Proboscis receptacle single-walled, slightly longer than 4 times as long as proboscis with cerebral ganglion at its base (Figs. 1, 3). Lemnisci long, digitiform, subequal, with 2 large oval giant nuclei each at widest part, reaching posterior end of anterior testis (Fig. 1).

**Figures 31–36:**
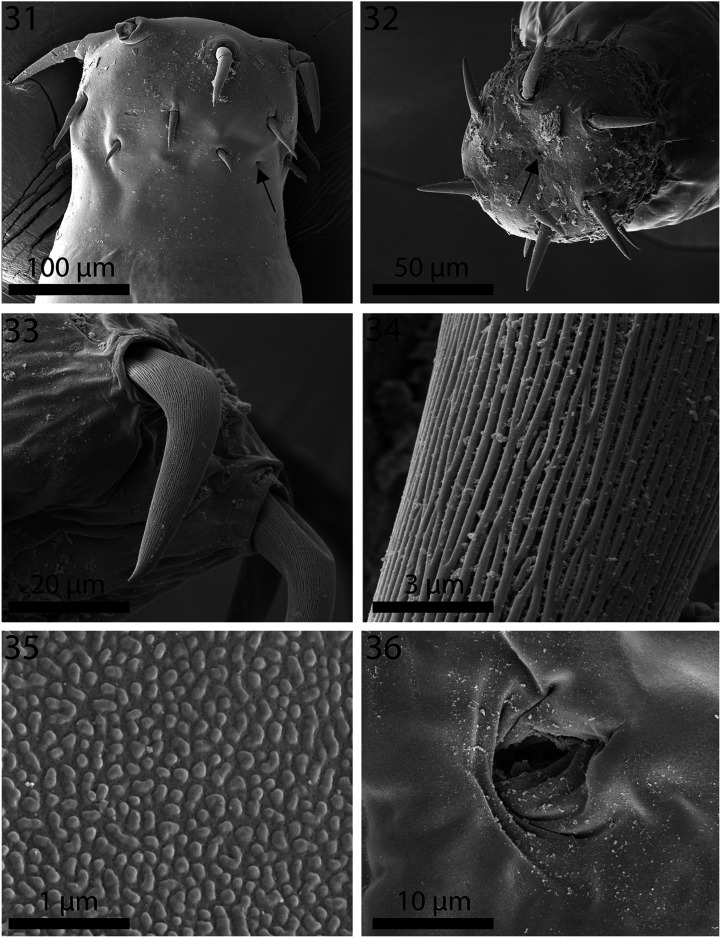
SEM of specimens of *Neoechinorhynchus ponticus* n. sp. from *Chelon auratus* from the Black Sea. **31.** The proboscis and neck of a male specimen showing the comparatively smaller hooks than in *N. personatus* and one of the neck sensory pores (arrow). **32.** An apical view of a proboscis showing the pore of the apical organ (arrow). **33.** The longitudinally serrated anterior proboscis hook of a specimen. **34.** Detail of the serration pattern on the hook shown in Figure 33. **35.** Detail of the pebbled surface of the neck of a specimen. **36.** Detail of the female gonopore.

*Male* (see Table 2 for measurements): Reproductive system in posterior two thirds of trunk. Testes large, equal, oblong, contiguous, slightly anterior to mid-trunk, with male reproductive structures extending into bursa. Cement gland longer than either testes, usually with 8–18 giant nuclei; mature adults with fewer nuclei. Cement reservoir just posterior to cement gland, less than half size of cement gland. Common sperm duct prominent. Saefftigen’s pouch prominent, overlapping common sperm duct and often obscured by it (Fig. 1). Bursa rounded, usually longer than wide, with no apparent sensory structures (Fig. 21). Gonopore terminal.

*Female* (see Table 2 for measurements). Reproductive system about 10% length of trunk (Fig. 3). Vagina not especially muscular. Uterus and uterine bell of moderate length with prominent ligament strand connecting anterior end of uterus at uterine glands with posterior body wall near the vagina dorsally (Fig. 6). Gonopore near terminal on ventral side. Eggs fusiform, elongate, with bluntly pointed ends and with polar prolongation of fertilization membrane (Figs. 5, 22).

#### Remarks

Morphologically, our specimens of *N. personatus* from Tunisia and the Black see were almost identical to those described by Tkach et al. [35]. The extent of the length of the lemnisci reaching the anterior testis, the shape and organization of the proboscis, hooks, and reproductive systems, and the number of giant sub-cuticular and cement gland nuclei were practically identical to those in the original description and to their Figures 12–18 (page 298). Our specimens, however, had slightly larger trunk, testes, cement glands and eggs, and slightly smaller hooks. References to the anterior trunk muscle ring, the ligament strand connecting the anterior end of uterus at the uterine glands with the posterior body wall, the micropores, and the neck’s sensory pores and pebble-like texture of its epidermis were missing from the original description. Characters used by Tkach et al. [35] to distinguish *N. personatus* from species of *Neoechinorhynchus* other than those in the *N. agilis* complex apply to the present discussion. The eggs in our specimens were slightly larger (35–50 × 10–15) compared to theirs (30–34 × 9–11). Egg size difference may be related to degree of development; their Figure 14 (mislabeled) shows 2 apparently underdeveloped eggs.

#### EDXA results for *N. personatus*

**Figure 37:**
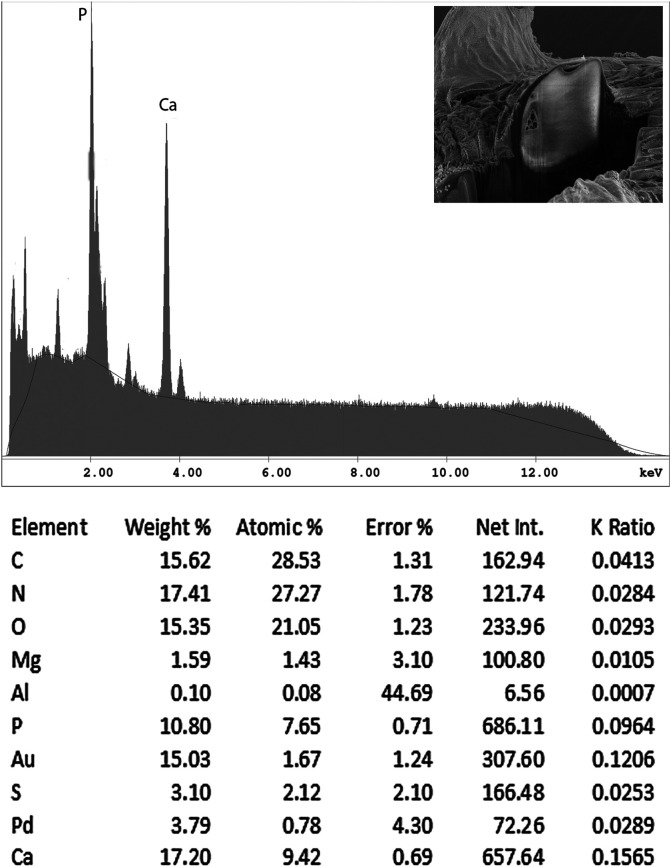
Energy dispersive X-Ray spectrum of the large anterior hook of a *Neoechinorhynchus personatus* specimen from *Mugil cephalus* in the Mediterranean showing high levels of phosphorus and calcium and low levels of sulfur. The X-ray data are the elemental analysis of the whole hook (see bolded figures in Table 3). Insert: SEM of a deep Gallium cut at the base of a hook.

**Figure 38:**
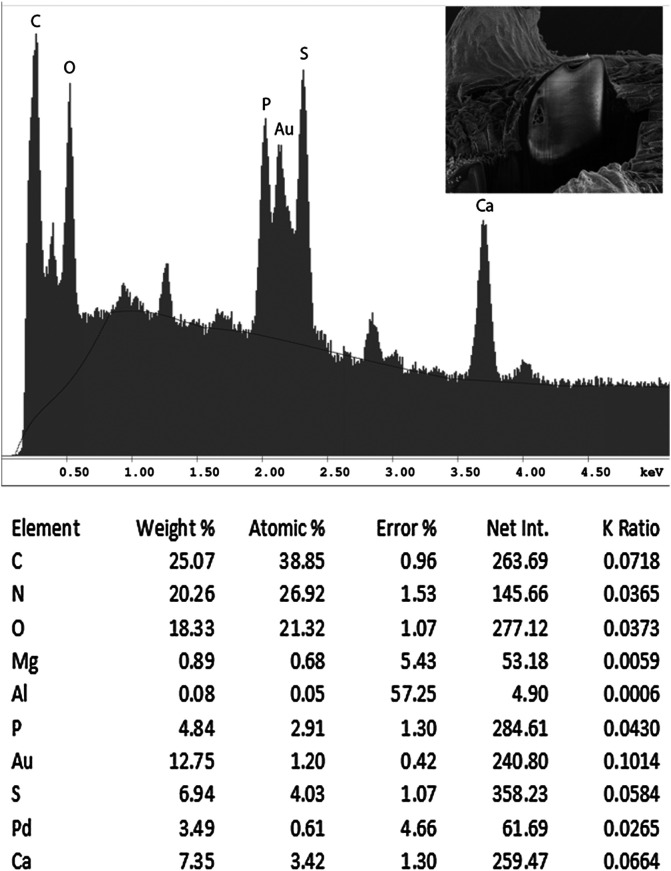
Energy dispersive X-ray spectrum of the small posterior hook of a *Neoechinorhynchus personatus* specimen from *Mugil cephalus* in the Mediterranean showing markedly lower levels of phosphorus and calcium, and considerably lower levels of sulfur than anterior hooks (Fig. 37). The X-ray data are the elemental analysis of the whole hook (see bolded figures in Table 3). Insert: SEM of a deep Gallium cut at the base of a hook.

Results for energy dispersive X-ray analysis are shown in Tables 3–6 and Figures 37, 38. The anterior and posterior hooks of our specimen of *N. personatus* from *M. cephalus* in both locations in the Mediterranean and the Black Sea had comparable biochemical profiles. All of the listed elements (P, S, Ca) varied for the two locations. Hooks from the Black Sea, however, exhibited higher levels of sulfur and lower levels of calcium (Table 3). The hardening elements (Ca, P) are more plentiful in the large anterior hooks. Table 3 also shows a difference in mineral content for the large and small hooks with small hooks showing higher sulfur and lower calcium levels (Figs. 37, 38 and Tables 3, 4). All elements were highest at the tip of hooks compared to the middle and bottom cuts (Table 5), and calcium was especially high at the base of hooks (Table 6). The hardening elements (Ca, P) are more plentiful in the large anterior hooks.

**Table 3:** Results of the X-ray microanalysis for elements present in the base of hooks of *Neoechinorhynchus personatus* from *Mugil cephalus* in two different geographical locations, the Mediterranean Sea off Tunisia and the Black Sea.

Mediterranean Sea off Tunisia	Large anterior hook		Small posterior hook	
	Wt%*	At%*	Wt%	At%
Phosphorus (P)	**10.80** **	7.65	**4.84** **	2.91
Sulfur (S)	**3.10**	2.12	**6.94**	4.03
Calcium (Ca)	**17.20**	9.42	**7.35**	3.42
Black Sea	Large anterior hook		Small posterior hook	
	Wt%	At%	Wt%	At%
Phosphorus (P)	8.54	6.2	2.69	2.12
Sulfur (S)	8.54	5.99	12.78	9.74
Calcium (Ca)	12.89	7.23	3.06	1.86

* Weight percent (Wt%) and Atom percent (AT%) are based on a total of 100 percent. Note: C, H, O, N are common elements in all protoplasm. Pd (palladium) and Au (gold) are used to count the specimen before viewing, Ga (gallium) is used to make fine cuts (dual beam) of specimens.

** See Figures 37 and 38 for EDXA spectra of bolded WT% of anterior and posterior hooks of *N. personatus* from the Mediterranean. Note that the levels of sulfur are about twice as high and the levels of calcium and phosphorous, relatively lower in all measurements (%) from specimens in the Black Sea than in those from the Mediterranean off Tunisia.

**Table 4:** Results of the X-ray microanalysis for elements present in the intact hooks of two species of *Neoechinorhynchus*, *N. personatus* from *Mugil cephalus* in the Mediterranean off Tunisia and *N. ponticus* from *Chelon auratus* in the Black Sea.

	Large anterior hook	Small posterior hook
	WT%*	WT%
*N. personatus*		
Phosphorus (P)	10.80	4.84
Sulfur (S)	3.10	6.94
Calcium (Ca)	17.20	7.35
*N. ponticus*		
Phosphorus (P)	8.54	2.69
Sulfur (S)	8.54	12.78
Calcium (Ca)	12.89	3.06

* Weight percent (WT%) is based on a total of 100. C, O, N are common elements of the protoplasm. Pd (palladium), Au (gold) and Ga (gallium) are omitted from the chart.

**Table 5:** Chemical composition of a small hook of *Neoechinorhynchus personatus* cut* at the three levels with a Gallium beam (LMIS) from a dual beam SEM.

Elements**	Tip cut		Mid cut		Base cut	
	Edge	Center	Edge	Center	Edge	Center
Magnesium (Mg)	0.34	1.94	1.99	1.82	3.34	1.89
Phosphorus (P)	9.38	19.04	15.13	19.10	12.13	18.64
Sulfur (S)	5.52	1.45	2.42	1.41	2.99	1.41
Calcium (Ca)	19.65	38.61	26.85	39.75	22.17	38.12

* Cross section cut.

** Common protoplasmic elements (C, N, O) and processing elements (Au, Pd, Ga) are omitted. Listed in WT%.

**Table 6:** Chemical composition of the base of large hooks for *Neoechinorhynchus* personatus and *N. ponticus* cut on a longitudinal axis with a Gallium beam (LMIS) from a dual beam SEM.

Elements*	*N. personatus*	*N. ponticus*
	Hook base	Hook base
Magnesium (Mg)	1.49	2.11
Phosphorus (P)	19.12	21.37
Sulfur (S)	1.33	1.43
Calcium (Ca)	54.75	45.00

* Common protoplasmic elements (C, N, O) and processing elements (Au, Pd, Ga) are omitted. Listed in WT%.

### *Neoechinorhynchus ponticus* n. sp. (Figs. 2, 4, 8, 10, 31–36)

urn:lsid:zoobank.org:act:F8CCB681-DDA9-4FD4-A240-54D57342646B

Type-host: *Chelon auratus* Risso (Mugilidae).

Type-locality: The Black Sea at Zalizny Port (46°7′ N, 32°17′ E).

Site of infection: Intestine.

Type-specimens: Harold W. Manter Lab collection no. HWML 102005 (holotype male and paratypes on same slide).

Etymology: The new species is named for the historical Greek name of the Black Sea.

The following description is based on the microscopic examination of 19 specimens of acanthocephalans (9 males, 10 females) from *Chelon auratus* in the Black Sea and on 6 more specimens examined by SEM. The supply of hosts limited the number of specimens available for this study. The description addresses mostly qualitative features that apply to specimens from this one host species in the Black Sea. Morphological and morphometric data are presented in Table 2. Biochemical, EDXA and molecular analysis are addressed separately, below.

#### Description

*General*: With characters of the genus *Neoechinorhynchus* as designated by Amin [1] (Neoechinorhynchidae). Medium-sized sexually mature adults. Shared structures larger in females than in males. Trunk somewhat enlarged anteriorly, gradually tapering at both ends (Figs. 2, 4). Body wall with electron dense micropores ducting into subcutaneous layer with multiple branching. Neck prominent with paired sensory pores (Fig. 31) and elevated pebble-like protrusions (Fig. 35). Body wall with prominent reticular lacunar system characteristically manifesting as heavily stained and lightly stained bands alternating at regular intervals, and with 5–7 (usually 6) dorsal and 1–3 (usually 2) giant hypodermal nuclei (Figs. 2, 4). Muscular ring at anterior trunk variable but often prominent (Fig. 8). Proboscis cylindrical, truncated and widest in anterior half, relatively but consistently wider than long, with prominent apical organ having 2 conspicuous nuclei at its distal end. Apical organ (Fig. 32) may reach or surpass level of posterior hooks, with 2 prominent giant nuclei (Fig. 8). All hooks with longitudinal serrations (Figs. 33, 34), rooted, most robust anteriorly, smallest posteriorly. Hook roots prominent but markedly shorter than blades, with anteriorly directed manubria. Manubria least prominent in largest anterior hooks (Fig. 10). Proboscis receptacle single-walled, slightly longer than 4 times as long as proboscis with cerebral ganglion at its base. Lemnisci of moderate size, digitiform, subequal, with 2 large oval giant nuclei each at widest part, falling far short of anterior testis and somewhat shorter in females (Figs. 2, 4).

*Male* (see Table 2 for measurements): Reproductive system in posterior half of trunk. Testes equal, equatorial to post equatorial, oblong, contiguous with male reproductive structures extending into bursa. Cement gland larger than either testes, usually with about 8 giant nuclei in adults but with up to 18 nuclei in juveniles. Cement reservoir just posterior to cement gland and less than half its size. Common sperm duct prominent. Saefftigen’s pouch prominent, about as long as cement gland, overlapping common sperm duct and often obscured by it (Fig. 2). Bursa usually longer than wide.

*Female* (see Table 2 for measurements). Reproductive system about 10% length of trunk, with prominent strand connecting anterior end of uterus at uterine glands with posterior body wall near vagina dorsally. Vagina not especially muscular. Uterus and uterine bell of moderate length, with terminal to near terminal gonopore (Fig. 36). Eggs not observed; only ovarian balls present.

#### EDXA results for *N. ponticus* n. sp.

**Figure 39:**
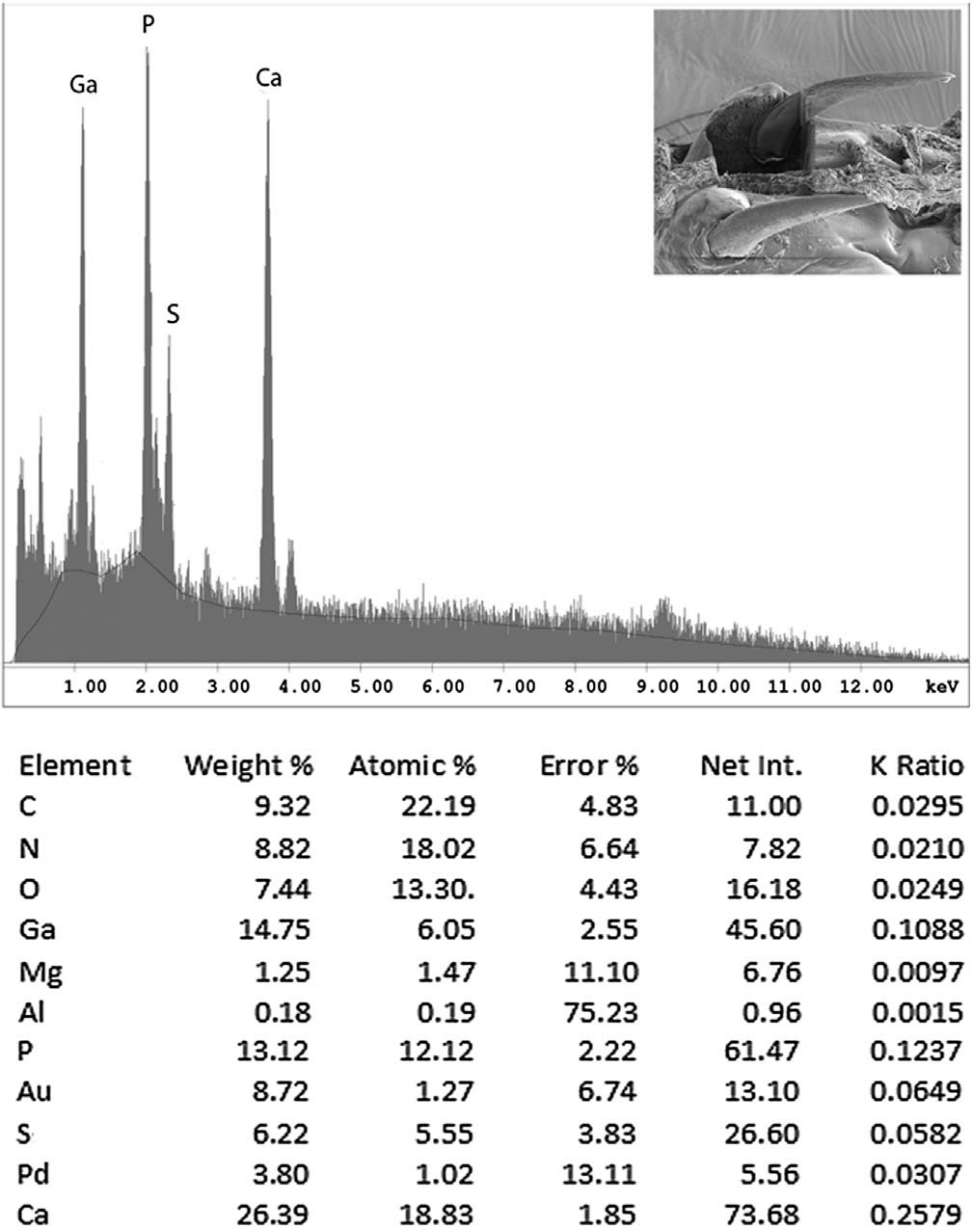
Energy dispersive X-ray spectrum of the tip of the anterior hook of a *Neoechinorhynchus ponticus* specimen from *Chelon auratus* in the Black Sea showing moderate levels of phosphorous, high levels of calcium, and moderate levels of sulfur. The X-ray data are the elemental analysis of the tip of the anterior hook (see bolded figures in Table 7). Insert: SEM of anterior hooks.

Tables 4, 6 and 7 and Figure 39 represent mineral (element) composition of the hooks of *N. ponticus.* Overall results are somewhat similar to those of *N. personatus* based on an average of the quantitative amounts, except that specimens of *N. ponticus* from the Black Sea exhibit higher levels of sulfur and lower levels of calcium (Tables 4, 6). The anterior large hooks have a marked difference in calcium (Ca) and sulfur (S) in comparison to the posterior small hooks (calcium 47.61 wt% versus 4.48 wt%; sulfur 2.99 wt% versus 36.42 wt%, respectively) (Table 7). These are figures from the base of hooks. Intact hooks showed the same pattern (Table 4). The base of the large anterior hooks appears to exhibit the highest level of calcium, as with *N. personatus* (Fig. 6).

**Table 7:** Weight percent (WT%) for three major elements* in cross section cuts of the anterior hook and the posterior hook of *Neoechinorhynchus ponticus* n. sp.

	Anterior hook			Posterior hook		
	Base (A)	Entry (B)	Tip (C)	Base (A)	Entry (B)	Tip (C)
	WT%	WT%	WT%	WT%	WT%	WT%
Phosphorus (P)	18.18	4.15	**13.12** **	17.47	13.36	1.77
Calcium (Ca)	47.61	65.20	**26.39**	4.48	39.65	36.42
Sulfur (S)	2.99	1.0	**6.22**	36.42	3.60	4.48

* Pd (palladium) and Au (gold) were used to count the specimens. Ga (gallium) was used for the cross cut of the hooks; other elements (C, O, N) are common in organic matter.

** See Figure 39 for EDXA spectrum of bolded WT% of tip of anterior hook of *N. ponticus* from the Black Sea. Inset: anterior hooks. Note that sulfur is much higher (36.42%) at the base of the posterior hook, calcium is highest (65.20%) at the entry of the anterior hook, and phosphorous is slightly higher (18.18%) at the base of the anterior hook.

#### Remarks

Tkach et al. [35] and Sarabeev et al. [31] satisfactorily differentiated 3 species in the *N. agilis* complex: *N. agilis* sensu stricto, *N. personatus*, and *N. yamagutii* from each other and from related species of *Neoechinorhynchus* Stiles and Hassall, 1905 outside of the *N. agilis* complex. The morphological features of our specimens from *M. cephalus* off the Tunisian Mediterranean and in the Ukrainian Black Sea are consistent with those of *N. personatus*, and so are the molecular profiles. Specimens of *N. ponticus* n. sp. from *C. auratus* in the Black Sea are clearly morphologically distinguished from those of *N. personatus* by the markedly smaller size of their trunk, proboscis, hooks, receptacle, lemnisci, testes and other male reproductive system structures (Table 2). In addition, the shorter lemnisci in *N. ponticus* occupy a considerably smaller trunk space distant from the anterior testis (Fig. 2) compared to usually overlapping with it in *N. personatus* (Fig. 1).

In *N. agilis*, the lemnisci are also distant from the testes, as in *N. ponticus,* but the roots of its anterior and middle hooks are much shorter in *N. agilis* with diminished manubria. In *N. ponticus* n. sp., the proboscis and proboscis receptacle are markedly longer while the hooks and testes are considerably smaller than in *N. agilis*; see Table 2 of Tkach et al. [35] and Table 2 (this paper). Specimens of *N. ponticus* lack the terminal papillae posterior to the gonopore characteristic of *N. agilis*.

The description of *N. yamagutii* by Tkach et al. [35] was heavily based on the description of *N. agilis* by Yamaguti [40] from *M. cephalus* in the Inland Sea and the Pacific coast of Mie Prefecture. Specimens of *N. yamagutii*, like those of *N. personatus*, have long unequal lemnisci reaching and overlapping the anterior testis (our Fig. 1 and Fig. 24 of Tkach et al. [35]) but they are larger worms with a considerably longer trunk (females reaching 25 mm) than *N. ponticus* and, unlike *N. ponticus* (Fig. 4), with decidedly subterminal female gonopore (Fig. 20 of Tkach et al. [35]). In *N. yamagutii*, the middle hooks are about half as long as the anterior hooks and barely longer than the posterior hooks. In *N. ponticus* n. sp., the transition in hook size from anterior is more gradual (our Table 2, and Table 4 of Tkach et al. [35]). In *N. ponticus*, the receptacle is larger but the testes and cement glands are considerably smaller than in *N. yamagutii*. In the description of *N. yamagutii* by Tkach et al. [35] references to Saefftigen’s pouch (1.00 long in Yamaguti [40]), or to the bursa (1.4 mm long in Yamaguti [40]) were missing.

### Molecular analysis

All *N. ponticus* n. sp. and *N. personatus* specimens successfully showed amplification of about 1300 bp for the partial 18S rDNA gene. The 18S rDNA dataset (1047 nt) included 25 sequences for different species of the genus *Neoechinorhynchus*, 3 *N. ponticus* n. sp. and 4 *N. personatus* sequences obtained in this study. Multiple alignment indicated that intra-species homology within *N. ponticus* n. sp. was 100%. Furthermore, pairwise distance illustrated the presence of an intra-species distance rate of 0–0.19% within *N. personatus* specimens obtained in the current study, and 0.18–0.67% compared to those with the sequences available in GenBank. Inter-generic differences based on partial 18S rDNA sequence between *N. ponticus* n. sp. with *N. personatus*, *N. agilis*, *N. yamagutii*, *N. dimorphospinus*, *N. buttnerae*, *N. crassus*, *N. pseudemydis*, *N. cylindratus*, *N. beringianus*, *N. saginata* and *N. simansularis* as other members of the genus *Neoechinorhynchus* were 3.24–4.49%, 3.26–3.69%, 3.84%, 3.49%, 5.06%, 5.45%, 14.91%, 4.55%, 4.02%, 5.12% and 4.02%, respectively. The sequence divergence based on the partial sequence of 18S rDNA between *N. personatus* with *N. ponticus* n. sp., *N. agilis*, *N. yamagutii*, *N. dimorphospinus*, *N. buttnerae*, *N. crassus*, *N. pseudemydis*, *N. cylindratus*, *N. beringianus*, *N. saginata* and *N. simansularis* was 3.24–3.76%, 2.18–2.78%, 6.22–6.60%, 7.11–7.34%, 10.21–10.46%, 10.45–10.72%, 14.42–14.70%, 9.60–9.86%, 10.32–10.60%, 10.13–10.39% and 10.32–10.60%, respectively.

**Figure 40:**
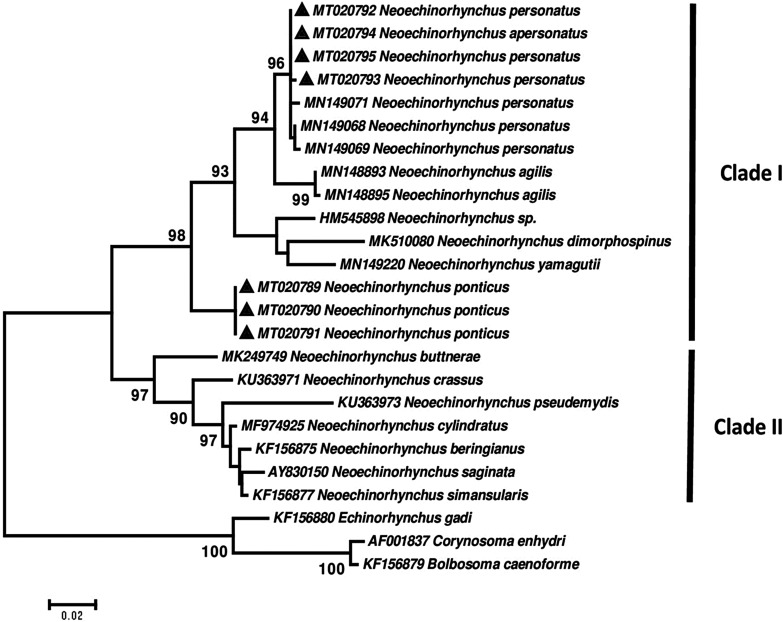
Phylogenetic tree of isolates of the two species of *Neoechinorhynchus* obtained in this study (▲) and other members of genus *Neoechinorhynchus* as retrieved from GenBank based on the partial 18S rDNA gene. The tree was constructed based on the maximum likelihood test and the Tamura 3-parameter model in MEGA6. *Echinorhynchus gadi*, *Corynosoma enhydri* and *Bolbosoma caenoforme* sequences were used as the out group. Bootstrap values lower than 70 were omitted.

The phylogenetic analysis illustrated two main clades. Clade I included species of *Neoechinorhynchus* associated with saltwater fishes, whereas clade II consisted of species from freshwater fishes. As shown in clade I, our sequences of *N. personatus* (MT020792 – MT020795) grouped with *N. personatus* isolates (MN149068, MN149069 and MN149071) from Ukraine with high statistical support. In addition, *N. agilis* (MN148893 and MN148895) clustered as a sister group of *N. personatus* isolates with high support. In addition, an unidentified species of *Neoechinorhynchus* (HM545898), *N. dimorphospinus* (MK510080) and *N. yamagutii* (MN149220) clustered as a sister group of the mentioned cluster with strong support. Our sequences of *N. ponticus* n. sp. (MT020789 – MT020791) are located at the basal position to the members of the *N. agilis* complex clade with 98% bootstrap support. Indeed, *N. buttnerae* (MK249749), *N. crassus* (KU363971), *N. pseudemydis* (KU363973), *N. cylindratus* (MF974925), *N. beringianus* (KF156875), *N. saginata* (AY830150) and *N. simansularis* (KF156877) clustered in clade II (Fig. 40).

### Electron dense micropores

Micropores were observed to be comparable in both species, *N. personatus* and *N. ponticus* in various trunk regions and were documented for specimens of *N. personatus* (Figs. 17, 20, 26, 29, 30). Unusual knobby epidermal surface of the neck (Figs. 17, 19, 26–28, 35) was also observed in both species.

## Discussion

### Morphometrics

None of the earlier descriptions of this variable acanthocephalan, *N. agilis*, accounted alone for its extreme morphometric variability. Host species and geography were not considered in the accounts given by Hamann [13], Linton [19–21], Van Cleave [36, 37], Meyer [23], Yamaguti [40, 41], Petrochenko [24], Gaevskaya et al. [10], Tepe & Oguz [34], and Tkach et al. [35], among others who described “*N. agilis*” from at least 10 species of hosts. Altogether, descriptions by the above cited authors gave an extreme range of variation in taxonomically important traits including trunk length of 7.00–45.00 mm, anterior, middle and posterior hook length of 70–150, 39–87, 20–71 respectively, proboscis receptacle 178–700 × 89–240, and testes 394–1820 × 97–570. Van Cleave [37] provided the most detailed description of the variability in *N. agilis* based on the examination of Rudolphi’s specimens from the Berlin Museum and the reports of the Dujardin, Stossich, Hamann, Condorelli, Porta, Monticelli, and Parona collections from Toulouse, Trieste, Rome, and Genoa (Table IV of Van Cleave [37]). Van Cleave [37] “found characterizations so diverse that it was difficult to determine whether the conflicting data represented individual variability within the species or resulted from inaccurate observations and erroneous identifications.”

### The species complex

None of the above accounts considered the possibility of dealing with a species complex. Tkach et al. [35] split *N. agilis* into three species (the nominal *N. agilis* and two new species) that overlapped in measurements of such important taxonomic traits as hook size and were collected from *M. cephalus* and other mugilids from various locations from the Mediterranean to Japan. Tkach et al. [35] collected one species, *N. personatus*, from *M. cephalus*, *C. auratus* and *Planiliza haematocheila* in the Mediterranean and in the Black Sea. Our specimens from *M. cephalus* in both locations proved to be *N. personatus* but those from *C. auratus* in the Black Sea turned out to be a different species, *N. ponticus* n. sp. The case for correlating taxonomic decisions with morphometric data, individual host species and geography was not clearly made by Tkach et al. [35]. These authors listed their *N. agilis* only from thick lipped grey mullet, *Chelon labrosus* Risso, leaving the type host “undefined” because of difficulties of ascertaining the host species from which Rudolphi’s [30] nine specimens were taken in Spezia, Italy (Type locality) as well as from the Bay of Biscay (Atlantic), and the Gulf of Santa Pola, Júcar Estuary (Mediterranean). It is worth noting that specimens of *N. agilis* identified by Linton [19] in the Atlantic off Wood’s Hole, Massachusetts, USA from the common eel, *Anguilla rostrata* Lesueur (Anguillidae), and the dusky shark, *Carcharhinus obscurus* Lesueur (Carcharhinidae), and in Fulton Market, New York from the white perch, *Roccus americanus* Gmelin (Moronidae) were correctly identified. Linton [19] did not provide a description, measurements or measurement bars of his whole male specimen (Fig. 70) that showed compatible proportions and disposition of the internal organs, including short lemnisci distant from the anterior testis characteristic of that species. This 130-year-old record alone expands the range of host and geographical distribution of *N. agilis*.

In our study, morphological differences noted in specimens from *M. cephalus* and *C. auratus* in the same location in the Black Sea (Table 2) correlate well with the designation of two different species of acanthocephalans supported by molecular data, *N. personatus* and *N. ponticus* n. sp., respectively. Measurements of key taxonomic characteristics of males and females of “*N. agilis*”, e.g. trunk, proboscis, hooks, testes, and eggs, from *C. auratus* on the Black Sea coasts of Turkey by Tepe and Oğuz ([34]; their Table 2) are very similar to those of *N. ponticus* n. sp. from the same host and body of water. We, therefore, assign the specimens reported by Tepe and Oğuz [34] to *N. ponticus* n. sp.

### Intraspecific morphological variations

Morphometric variations in specimens of *N. personatus* from the same host species, *M. cephalus*, in the Mediterranean and from the Black Sea (Table 2), would point to intraspecific geographical variations. Van Cleave [37] made a vague reference to “geographical varieties” that was not supported by hard data. The observed variations are comparable to the more dramatic case of *Mediorhynchus papillosus* Van Cleave, 1916 (Gigantorhynchidae) featured by Amin and Dailey [2]. These authors studied key taxonomic characteristics in various geographical populations of *M. papillosus* which has a wide range of distribution in at least 73 species of birds outside of North and South America in Asia from Taiwan to the east into China, many of the former Soviet Republics, and to Eastern Europe to the west. Amin and Dailey [2] demonstrated a distinct geographically-based variability, especially in the size of proboscis and its armature, neck, receptacle, and testes, that appeared related to geographical restrictions, intermediate and definitive host specificity and distribution, and host feeding behavior. We recognize that many fish hosts of species of *Neoechinorhynchus* are migratory and that their feeding grounds and specimen collecting localities may not always be the same. However, this situation is also comparable to that of migratory bird hosts of *M. papillosus.*

### Electron dense micropores

Micropores are present throughout the epidermal surface of the trunk of species of *Neoechinorhynchus*, like those reported in other species of the Acanthocephala, are associated with internal crypts, and vary in diameter and distribution in different trunk regions corresponding with differential absorption of nutrients. We have documented this phenomenon in 16 species of acanthocephalans [16] and a few more since. The functional aspects of micropores in a few other acanthocephalan species including *Rhadinorhynchus ornatus* Van Cleave, 1918, *Polymorphus minutus* (Goeze, 1782) Lühe, 1911, *Moniliformis moniliformis* (Bremser, 1811) Travassos (1915), *Macracanthorhynchus hirudinaceus* (Pallas, 1781) Travassos (1916, 1917), and *Sclerocollum rubrimaris* Schmidt and Paperna, 1978 were reviewed earlier by Amin et al. [3]. We demonstrated the tunneling from the cuticular surface into the internal crypts by TEM (Figs. 26, 29, 30). Wright and Lumsden [39] and Byram and Fisher [8] reported that the peripheral canals of the micropores are continuous with canalicular crypts. These crypts appear to “constitute a huge increase in external surface area … implicated in nutrient uptake.” Whitfield [38] estimated a 44-fold increase at a surface density of 15 invaginations per 1 μm² of *Moniliformis moniliformis* (Bremser, 1811) Travassos, 1915 tegumental surface. The micropores and the peripheral canal connections to the canaliculi of the inner layer of the tegument of *Corynosoma strumosum* (Rudolphi, 1802) Lühe, 1904 from the Caspian seal *Pusa caspica* (Gmelin) in the Caspian Sea were demonstrated by transmission electron micrographs in Amin et al. [4] and in the present material from *N. personatus* from *M. cephalus* in Tunisia (Figs. 26, 29, 30). We cannot explain the nature or functionality of the pebble-shaped texture of the neck in specimens of these two acanthocephalan species from either *M. cephalus* or *C. auratus*. We can, however, use this unique feature as a diagnostic tool for these two species. We do not know if the necks of other species of the *N. agilis* complex are similar. We do know that other species of *Neoechinorhynchus* that we have studied did not possess this feature.

### Energy dispersive X-ray analysis

Our studies of acanthocephalan worms have usually involved X-ray scans (EDXA) of gallium cut hooks and other worm structures [14, 15, 33]. Both large and small hooks were evaluated for chemical ions, with sulfur (S), calcium (Ca) and phosphorus (P) being the prominent elements. Sulfur as expected, was high especially at the outer edge of large hooks of specimens from the Black Sea. Calcium and phosphorus are major ions at the base and middle of the hooks (Table 3). There was a difference in chemical content for large and small hooks. Large hooks play a major role in host tissue attachment, which may account for the difference. The anterior and posterior hooks of our *N. personatus* from *M. cephalus* in the Mediterranean and Black Sea had comparable biochemical profiles, especially for phosphorous. Variable levels of sulfur and calcium in hooks between the Mediterranean and the Black Sea specimens (Table 3) may be attributable to different levels of accumulation of metals in the two bodies of water. It should be noted, however, that extreme differences may occur in different parts of the same hook of acanthocephalan species found in the same host at a single location, or between anterior vs. posterior hooks on the same proboscis. The levels of sulfur were markedly higher in *N. ponticus* from the Black Sea compared to *N. personatus* from the Mediterranean which may also be attributable to the rich resources of sulfur in the Black Sea [9]. For instance, in *Cavisoma magnum* (Southwell, 1927) Van Cleave, 1931 from *Mugil cephalus* in the Arabian Sea, unusually high levels of sulfur in hook tips (43.51 wt%) and edges (27.46 wt%) were found. The center and base of hooks of the same worms had negligible sulfur levels and contained mostly phosphorus and calcium, the two other essential elements for hook structure [5]. Like fingerprints, the EDXA appear to be species-specific and has significant diagnostic value in acanthocephalan systematics [6]. For example, *Moniliformis cryptosaudi* Amin, Heckmann, Sharifdini, and Albayati, 2019 was erected based primarily on its EDXA pattern [6]. Our methodology for the detection of the chemical profile of hooks in the Acanthocephala has also been used in other parasitic groups including the Monogenea [27, 29] and Cestoda [28]. We also provide chemical and molecular data to explain and clarify our findings.

### Molecular analysis

In the last few decades, a PCR-sequencing technique was applied for assessment of taxonomic identification, diversity and phylogenetic relationships among acanthocephalan species [11]. Inter-generic differences are noted between *N. ponticus* n. sp. and *N. personatus* with other members of the genus *Neoechinorhynchus* based on partial 18S rDNA being 3.24–14.91% and 2.18–14.70%, respectively. The relatively high genetic differences between *N. ponticus* n. sp. and other species of the *Neoechinorhynchus* supports the morphological observation indicating it is an independent species. Sarabeev et al. [31] showed that *N. agilis*, *N. personatus* and *N. yamagutii* are three independent species using the same phylogenetic analysis of partial sequences of the 18S rRNA gene. Our data, consistent with results of other studies [22, 31], indicated genetic divergences among these three species were relatively high, at the range between 2.18% and 6.60%. The phylogenetic analysis of the partial 18S rDNA gene illustrated that our sequence of *N. personatus* clustered with *N. personatus* isolates from Ukraine. Genetic differences between *N. agilis* and *N. personatus* were found to be low. Moreover, the tree also showed they are two closely related species. Our sequences of *N. ponticus* n. sp. were placed separately from other species of *Neoechinorhynchus* in clade I with strong support. The position of *N. ponticus* n. sp. along with other species of *Neoechinorhynchus* from saltwater fishes including *N. dimorphospinus, N. yamagutii, N. agilis* and *N. personatus* in the tree, indicated that all these species share a common ancestor. This would make *N. dimorphospinus* a candidate for membership in the *N. agilis* complex (see Fig. 40) especially that specimens of *N. dimorphospinus* are morphologically comparable to the 4 species of the *N. agilis* complex. It is also grouped with them in one major clade with 93% similarity with the Tunisian *N. personatus*. However, *N. dimorphospinus* is distinguishable morphologically by having the 2 lateral proboscis hooks in the anterior ring being considerably longer than the other 4 hooks in the same ring. It has been found in 6 species in 4 families of marine fish in the Persian Gulf and in the Pacific Ocean off Vietnam. In species of *Neoechinorhynchus*, high level of variation in the *cox1* gene would provide better resolution of the relationships within closely related taxa [25]. Due to the lack of sufficient sequences of *cox1* sequences of *N. agilis* complex in GenBank for comparison, we did not perform molecular analysis of these specimens with this gene. Pinacho-Pinacho et al. showed that the *cox1* gene is an informative molecular marker compared to the ITS and 28S rDNA genes for detection of cryptic species complexes in *Neoechinorhynchus* in Middle America [25]. However, their analysis is for the species inhabiting fresh and brackish water fish across Middle America, a different zone from our study areas. Providing additional genetic markers as well as obtaining more specimens of these species from different geographical locations and hosts would be useful to better understand their phylogenetic relationships.
